# Strengthening systems of care for people with or at risk for HIV, HCV and opioid use disorder: a call for enhanced data collection

**DOI:** 10.1080/07853890.2022.2084154

**Published:** 2022-07-01

**Authors:** Anthony J. Eller, Elizabeth E. DiDomizio, Lynn M. Madden, Jennifer D. Oliva, Frederick L. Altice, Kimberly A. Johnson

**Affiliations:** a Section of Infectious Diseases, Department of Internal Medicine, Yale University School of Medicine, New Haven, CT, USA; b Department of Mental Health Law & Policy, University of South Florida, Tampa, FL, USA; c Center for Health & Pharmaceutical Law, Seton Hall University School of Law, Newark, NJ, USA

**Keywords:** Opioid use disorder, HCV, HIV, cascade of care, continuum of care, implementation

## Abstract

**Background:**

The syndemic between opioid use disorder (OUD), hepatitis C virus (HCV), and human immunodeficiency virus (HIV) results in excessive burdens on the healthcare system. Integrating these siloed systems of care is critical to address all three conditions adequately. In this implementation project, we assessed the data capacity of the health system to measure a cascade of care (COC) across HIV, HCV and OUD services in five states to help guide public health planning.

**Materials and methods:**

Data for this study were gathered from publicly available datasets and reports from government (SAMSHA, CMS, HRSA and CDC) sites. We created, where possible, COCs for HIV, HCV, and OUD spanning population estimate, diagnosis, treatment initiation, treatment retention, and patient outcomes for each of five states in the study.

**Results:**

The process of data collection showed that baseline COCs examining the intersections of OUD, HIV, and HCV cannot be produced and that there are missing data in all states examined. Collection of specific data points is not consistent across all states. States are better at reporting HIV cascades due to federal requirements. Only gross estimates could be made for OUD cascades in all states because data are separated by payer source, leaving no central point of data collection from all sources. Data for HCV were not publicly available.

**Conclusion:**

It is difficult to assess the strategies needed or the progress made towards increasing treatment access and decreasing the burden of disease without the ability to construct an accurate baseline. Using integrated COCs with relevant benchmarks can not only guide public health planning, but also provide meaningful targets for intervention.

**KEY MESSAGES**
- While HIV COCs are available for most states at least annually, they are not disaggregated for populations with co-occurring OUD or HCV.
- Data to calculate HCV COC are not available and data to calculate OUD COC are partially available, but only for specific payers.
- States do not have systems in place to measure the scope of the syndemic or to identify targets for quality improvement activities.

## Background

The volatile opioid epidemic has resulted in unprecedented morbidity and mortality, including overdose, hospitalisation, and new outbreaks of HIV and HCV in nearly every state. With ∼2.2 million Americans having an opioid use disorder (OUD), overdose deaths reached an unprecedented high of 94,360 in 2020 with a 31% increase occurring in a single year [1,2]. In parallel, there are ∼1.2 million people with HIV (PWH) [3], with 7% of the 36,801 newly reported HIV cases in 2019 being among people who inject drugs (PWID) and over 2.4 million people living with active HCV infection [4]. From 2009 to 2018, the number of reported acute Hepatitis C cases per 100,000 population increased threefold from 0.3 to 1.2 [5]. Research evidence suggests that much of the growth in Hepatitis C infections is related to injection drug use and that treating OUD would reduce the global burden of HCV as well as HIV [6–9]. The confluence of these three diseases, especially among those most disenfranchised from the healthcare system (e.g. homeless, food insecure, impoverished, minority), has emerged as an especially challenging syndemic.

A syndemic is defined as the convergence of two or more diseases combined with a social factor such as poverty, race, incarceration or stigma that mutually reinforces the negative consequences of the disease conditions [10,11]; syndemic conditions are crucial for understanding the social determinants of health. Syndemics are most effectively addressed by attacking the root causes using evidence-based services. Policies and siloed funding streams often constrain effective implementation of effective strategies. The toolkit of evidence-based interventions to prevent HIV transmission in people who inject drugs serves as a fulcrum to address the OUD/HIV/HCV syndemic. The HIV prevention toolkit for people who inject drugs has evolved over the course of forty years to include – syringe services programs (SSP), pre-exposure prophylaxis (PrEP), HIV treatment as prevention (TasP), and medications for opioid use disorder (MOUD) [12,13]. One policy analysis of 11 public health or policy strategies to reduce death due to the opioids epidemic found MOUD to be among the most effective strategies to reduce death [14]. MOUD also has the potential benefit to impact the HIV epidemic. For example, of the four evidence-based practices to reduce HIV transmission, MOUD is the single most likely strategy to have the greatest impact on all three conditions (see Table 1), although a combination of all available interventions is needed for the greatest impact. MOUD has demonstrated the improvement of the HIV and HCV treatment and prevention cascades [15,16] in addition to the 60** **years of evidence for being the best treatment for OUD [17]. Despite the evidence for MOUD, its scale-up has been hindered in part due to structural impediments including the disconnect between service provision and funding for OUD, HIV and HCV [18].

**Table 1.** Evidence-based toolkit for syndemic interventions.

	MOUD	SSPs	PrEP	TasP
HIV	✓	✓	✓	✓
HCV	✓	✓		
OUD	✓			
Social instability	✓			
Mortality	✓		✓	✓

A checkmark within a cell indicates the effective contribution of a particular treatment intervention towards prevention of the outcome in the left-most column.

To address these inter-related epidemics, it is crucial to understand gaps and opportunities for intervention. Care continuums for HIV, HCV and OUD provide a heuristic to identify targets for intervention and to assess effectiveness of strategies deployed. A care continuum or cascade of care can identify where in the process from identification through treatment outcomes a population health strategy would have the largest effect by identifying gaps in the process of care at a population level.

In fall 2019, the Health Services Resources Administration (HRSA) as part of their Special Projects of National Significance (SPNS) tasked investigators to support five states in implementing projects to improve systems of care for people with or at risk of OUD, HIV and HCV in two regions with high rates of OUD and HCV and HIV outbreaks (New England and Appalachia). This project included a landscape analysis of the current state of service utilisation using a cascade of care (COC) model so that (1) high yield opportunities along the care continuums and services delivery systems could be identified and (2) effects of the interventions could be measured. The goal was to first establish the COC for each condition and define their overlap to identify the extent of the intersections. This baseline was to be used to identify opportunities for targeting implementation of evidence-based interventions. Findings from this landscape analysis are presented along with potential opportunities for improvement.

## Materials and methods

### States

Funding for this study was provided by HRSA, which funded a landscape analysis of five diverse states that differed in terms of their HIV and opioid epidemics, the array of available services and funding streams, geographical diversity (urban, suburban, rural) and experiences with coordination.The populations estimates found in Table 2 provide contextual background for the states examined.

 .

**Figure 1.**
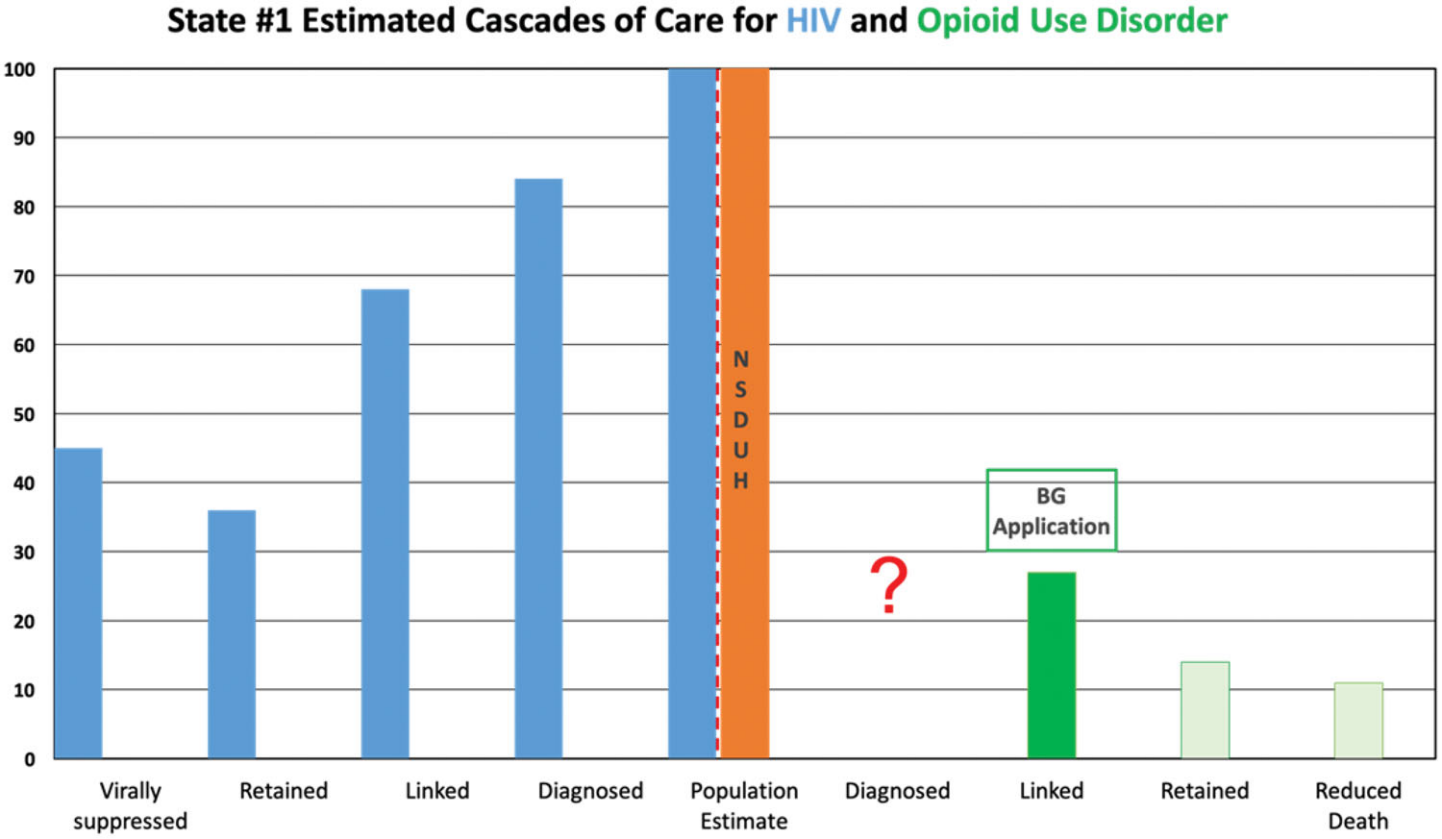
State #1 cascades of care estimates. Axis definitions – “Linked” is equivalent to “linked to care”. “Retained” is equivalent to “retained in care”.

### Data definitions

As part of the landscape analyses, we first created a list of definitions to describe the COC for each condition and then began collecting data from publicly available resources utilising a standard methodology. The intent was to construct a COC for HIV, HCV, and OUD and the intersection between the three to assess to what extent that states were responding to these three intertwined epidemics.

#### Continuum of care elements

##### HIV

Over the past decade, the HIV COC has been refined to include four elements that can be measured as quality care outcomes and are included in the UNAIDS 95-95-95 strategy. These include: (1) a population estimates; (2) identification/diagnosis, (3) antiretroviral therapy (ART) receipt; and (4) viral load suppression. Others have also included linkage to and retention in care, sometimes referred to as treatment engagement, into this COC. Where possible, we include as many elements as are available in HIV surveillance databases. These universal measures along with a simply expressed set of targets (the 95-95-95 goals) have led to international success in reducing the burden of HIV [19,20].

##### HCV

For HCV, the population estimates have changed appreciably as scale-up of treatment has evolved using direct acting antivirals (DAAs), which unlike the case for HIV, cure HCV infection (though re-infection can occur). The core elements of the HCV COC include: (1) population estimates; (2) HCV treatment; (3) cure; and (4) reinfection. Reporting systems in most states, however, are not designed to address this cascade, making it challenging to approach it from a syndemic perspective. As HCV is mostly transmitted through drug injection, scant data are available about the type of drug (i.e. opioids, stimulants) that is involved in HCV transmission.

##### OUD

Though there has been considerable work to create a COC for infectious diseases like HIV and HCV, only recently have researchers and policy makers begun to create a similar COC for OUD. Consequently, there are little data collected that help to overcome this as such heuristics could greatly guide implementation and scale-up [21,22]. For this analysis, we define five key elements that include: (1) population estimates; (2) identification/diagnosis; (3) linkage to MOUD treatment; (4) retention in MOUD treatment; and (5) reduced death [23].

### Data sources

Because the aim of the project was to increase use of MOUD and because HIV cascades were readily available, for the most part, from states due to Ryan White support, we focussed data collection on developing OUD COCs. We defined the population estimate as the prevalence of OUD using the Substance Abuse and Mental Health Services Administration’s (SAMHSA) National Survey on Drug Use and Health (NSDUH) variable for meeting Diagnostic and Statistical Manual of Mental Disorders, 5th Edition (DSM-V) diagnostic criteria for OUD in the prior 12** **months. All states’ population estimates for OUD were calculated using NSDUH, though other calculations are also included for the two states that had their own estimates; the NSDUH estimates are low due to large numbers of people with OUD in settings (e.g. prisons/jails/hospitals/homeless persons) who are not housed and therefore not included in the household survey. We defined treatment as receiving MOUD – either methadone or buprenorphine. MOUD is highly regarded as the most effective OUD treatment for preventing opioid craving, relapse, and overdose [24,25]. When comparing methadone and buprenorphine with other common treatment modalities – opioid antagonist therapy, inpatient treatment, or intensive outpatient behavioural interventions – only MOUD was associated with reduced risk of overdose and reduction in hospitalisation during 3 and 12-month follow-up [26]. The percent of the population receiving a diagnosis was not available in any state. Treatment entry was obtained from either the state, or from the Treatment Episode Data Set online analysis tool from SAMHSA [27]. It is reported as the number of new admissions in the calendar year. Treatment retention data were uniformly unavailable, as are outcomes related to treatment. All data sources can be found in Table 3.

### Data collection process

In an attempt to locate better data for estimates of the opioid COC, we developed a standardised process of searching publicly available data sources for each state. We used a multi-step search process to glean public sources for information that could describe access to, retention in, and outcomes for treatment of HIV, HCV and OUD. The first step in the process was to find existing reports that included a COC for any of the three conditions. If they did not exist, we attempted to create COCs using publicly available data. No public sources of data other than incidence rates could be found on HCV and thus, we ended our search and did not attempt to create COCs for HCV. Most states have HIV information web pages as part of their infectious disease division websites, where HIV cascade of care data are usually reported at least once a year (annual report).

For creating the OUD COC, we reviewed state websites with a focus on substance abuse authority and public health authority pages to locate reports or live data analysis tools. Some states are making de-identified data available for analysis online, which makes it easier to ascertain the number of people using specific medications. These data also can show the frequency of co-infections, the medications that were used for treatment, and individual characteristics like age, race and gender among the population. In the absence of data, we specifically sought out the following reports on state substance abuse pages: overdose reports, treatment utilisation reports, planning documents including federal Substance Abuse Prevention and Treatment (SAPT) block grant or State Targeted Response (STR) and State Opioid Response (SOR) grant proposals.

If data were not available on the state website to develop each COC, we searched federal websites such as SAMHSA, Centres for Medicare & Medicaid Services (CMS), HRSA and Centres for Disease Control and Prevention (CDC) to locate data or reports on specific states. Information and data were obtained from grant abstracts and federal reports that included information about the barriers to treatment access within a state. Federal reports provided context and funding information that might also be missing from state government websites.

We resorted to using the SAMHSA’s NSDUH Public Use Data Analysis System (PDAS) and its Restricted Use Data Analysis System (RDAS) [28] for prevalence for all states. For all states, we ran estimates from the 2016/2017 NSDUH on OUD prevalence and treatment utilisation from the NSDUH two-year RDAS online analysis tools. We also ran an analysis on the Treatment Episode Discharge Data (TEDs-D) for 2017 which was the most recent year available to calculate the percent of publicly funded patients in treatment for OUD that had received medication. We reviewed other sites that have national or state comparison reports or data analyses that might have useful but slightly old information (e.g. Kaiser, Urban Institute, Rand, McKinsey). We searched for recent (since 2016) peer review publications for data on HIV or OUD in the state with search terms that included the state name and the condition name. For any information that might have still been missing, we conducted an internet search of the state name and whatever data element we concluded was missing at that point (For example: *STATE* OUD treatment utilisation). Despite efforts to find either COCs or better data to develop them, for most of the states, we used NSDUH for population estimates of prevalence, TEDs for treatment entry, and the peer review literature for averages for treatment retention and treatment outcomes. Exceptions to this methodology are noted in the graphs of the COCs in the results section.

After having conducted the searches, we met with state stakeholders to review what we had found and to determine whether our search process had missed anything. We also inquired if they had data that were not available publicly to create the COCs. Only two states had additional data to better inform our COCs. Only COCs reviewed by or updated by state stakeholders are reported.

## Results

### HIV continuum of care

Three of the five states had publicly available reports which included data on the COC for HIV. Two of the five states did not have publicly available data related to the HIV COC but provided the necessary continuum data upon request. None of these cascades, however, were able to be stratified by HCV and/or OUD status. States had data on the percent of people with newly diagnosed HIV that had acquired their infection *via* drug injection, but did not have information on whether those people fared differently in treatment.

### HCV continuum of care

While all states had HCV prevalence and incidence data, none reported on the other measures in the COC and data were not readily available in any other location, thus cascades could not be constructed. Challenges we incurred were that there was no central database for the surveillance of treatment information because HCV data are only collected in medical records and claims data, so it was extremely difficult to attempt to gather all of the information we needed. In states where HCV is reportable, most sites indicated that the data were incomplete and generally did not have any treatment outcome data, including cure.

### OUD continuum of care

There have been recent calls to utilise an OUD COC [21,29]. None of the states had a COC for OUD publicly available in reports or data that could be shared or accessed by the work group participants. It was necessary to build multiple estimates for OUD because data are collected by various funders and one source may have very different COCs depending on what population was included to collect data from.

#### Population estimate of OUD

OUD prevalence was measured using NSDUH, considered by many in the research community to be a two to four-fold undercount of actual cases due to the sampling framework for NSDUH [30]. Two states had their own database with population estimates that indicated a much higher prevalence. For those two states, we share both estimates in this paper to understand the contrast between federal government and state government data surveillance.

#### Diagnosis

No state provided publicly available data on state-wide verified diagnosis of OUD.

#### MOUD treatment entry

Estimates for treatment entry came from either a state block grant application (one state had specific numbers), a state Medicaid report, a state monthly opioid update, or TEDS admission data. The value of the TEDS admission data varies by state depending upon what they report, as states often have different reporting mechanisms. For example, one state reports data on all patients that receive treatment in a program that receives public funding, while another reports only on those patients whose treatment is covered by the Substance Abuse Prevention and Treatment Block Grant (SAPTBG) funding. Some states have very distinct public and private systems (e.g. Florida and California) and others have one system with multiple funding streams (e.g. Vermont and Maine). Data from the available sources provided widely varied results, so for two of the five states, we provided a range of possible values for the OUD treatment COC.

#### Retention

Treatment retention data were unavailable from any state; however, upon request, one state did provide an average length of stay from their Medicaid data, but were not able to report data on the threshold of 180** **days. Of note, for many settings, Medicaid covers approximately half of MOUD provision. Therefore, we used a retention estimate of 50% after 180** **days based on research [23,31,32].

#### Combined continuum of care

**Figure 2.**
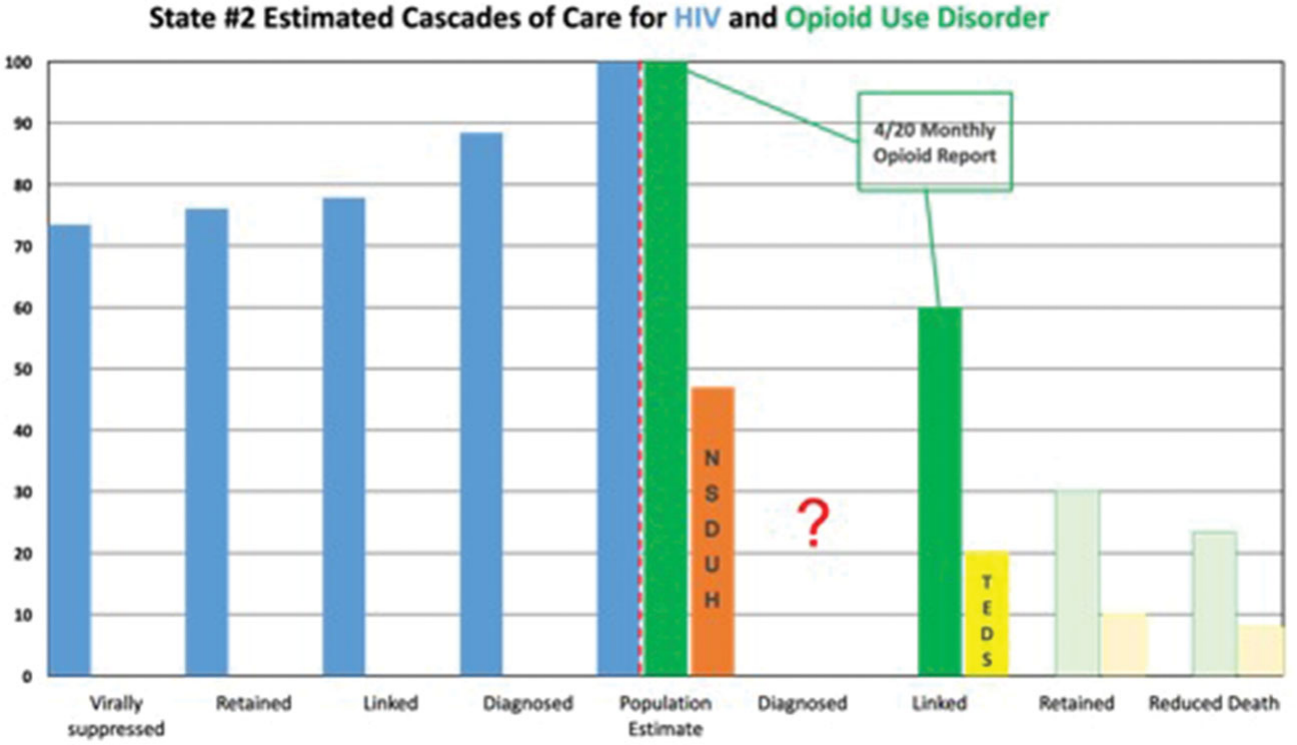
State #2 cascades of care estimates. Axis definitions – “Linked” is equivalent to “linked to care”. “Retained” is equivalent to “retained in care”.

**Figure 3.**
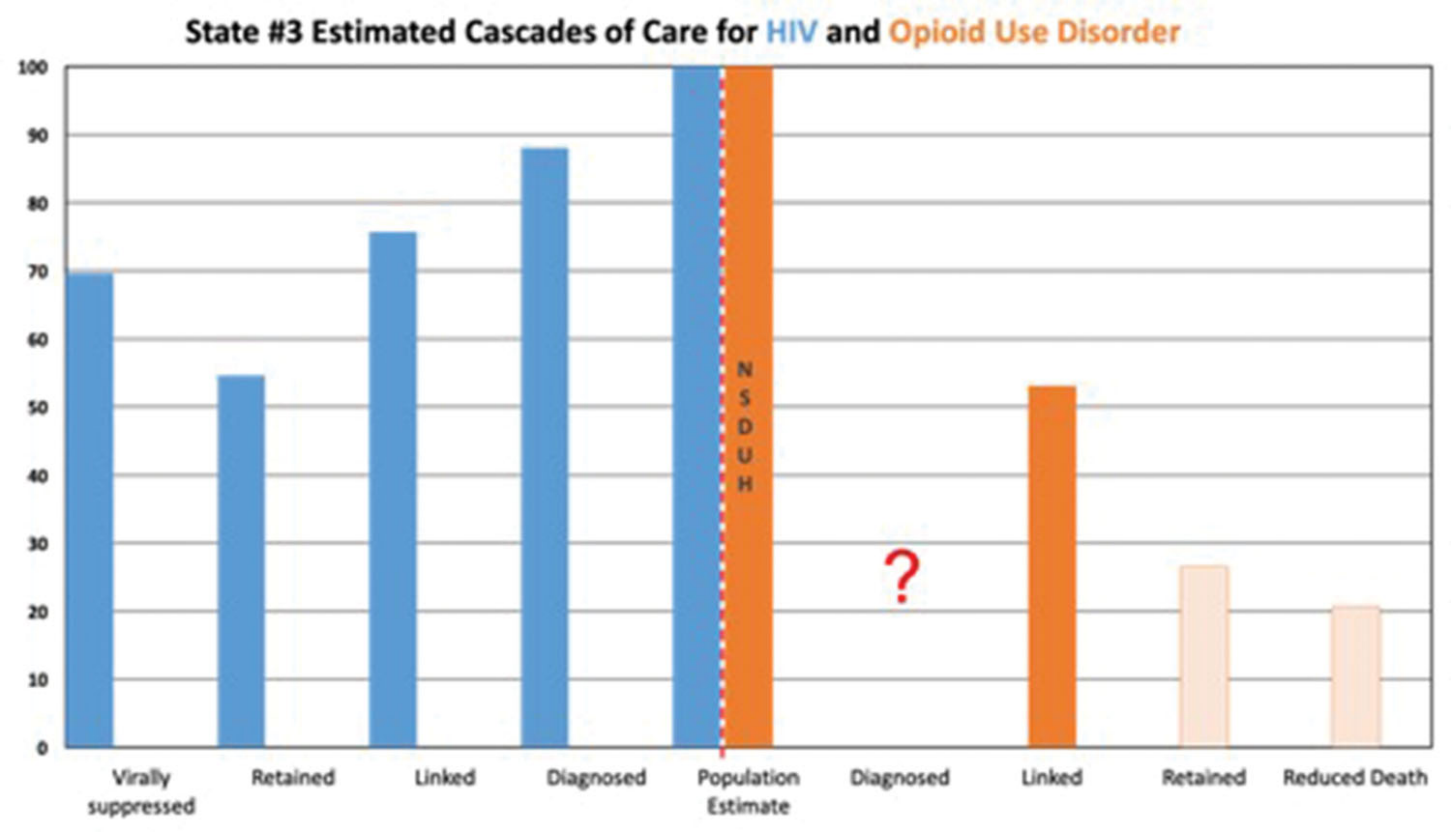
State #3 cascade of care estimates. Axis definitions – “Linked” is equivalent to “linked to care”. “Retained” is equivalent to “retained in care”.

**Figure 4.**
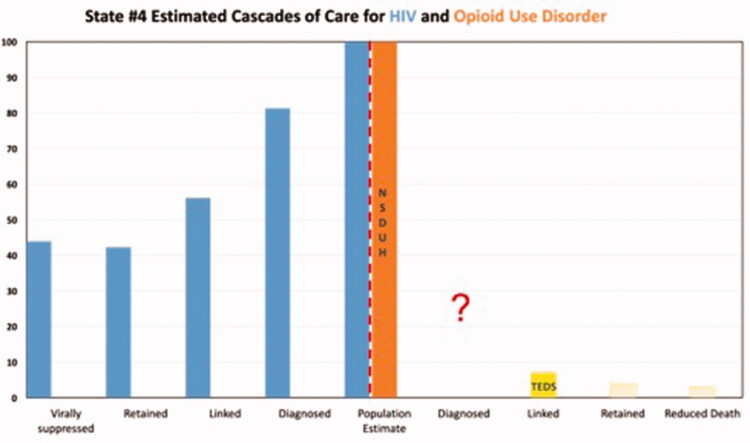
State #4 cascade of care estimates. Axis definitions – “Linked” is equivalent to “linked to care”. “Retained” is equivalent to “retained in care”.

Results are depicted graphically in Figures 1–5. No state had an integrated COC for people who had both HIV and OUD. We were unable to use existing data to create a combined COC for people co-infected with both conditions, or to include HCV in any of the analyses. Each state indicated that a combined COC would be helpful for planning.

Each state estimate was calculated from different data sources and therefore these data are not comparable across states. As population sizes are widely different, note the difference in scale for each graph. Despite the significant data issues, it is clear from the charts below that in most cases, linkage to care is the primary target for states and communities trying to improve their treatment systems for OUD, where retention in care is the target for HIV. The OUD COCs have so many limitations, that they would not be useable for our original intent of measuring the impact of system-level quality improvement activities.

## Discussion

To our knowledge, this is the first attempt at utilising a cascade of care approach to overlay and depict the syndemic of HIV, HCV, and OUD across multiple states in the United States. The intention of this methodology was for states to use the COCs constructed in this analysis to plan effective interventions and measure improvement in systems functioning for these interlinked conditions. Based on the findings, this analysis provides evidence that with the current incomplete, disparate, and non-integrated data systems across states, it is not feasible to construct all cascades, therefore, interfering with adequate public health policy planning. Given that MOUD is the most promising strategy for addressing all three conditions simultaneously [33], the inability to construct accurate OUD cascades in particular undermines effective implementation efforts to address this syndemic.

The absence of data systems that track OUD and HCV at a population-level means it is not possible to construct baselines to accurately depict the population-wide treatment needs of individuals with either or both conditions, as they are known to overlap. Moreover, the inability to construct complete COCs for any of the three conditions inhibits implementation of targeted interventions to address a specific step in the cascade. The finding that MOUD not only reduces primary HIV and HCV transmission [17], but also improves each step of the HIV COC, including reduced dropout [16], supports the need for accurate COCs to guide more effective implementation.

While the methods we deployed for creating the OUD cascade might be useful, the variance in data systems across states precludes comparison of outcomes. Regarding OUD treatment outcomes, the values are dependent upon each state’s particular TEDS data system and the percent of the treatment population captured. Based on the states included in this study, it is clear that there are differences across payer and data source. While some states have created all-payer claims databases, the states in our study either do not have them or do not use them for decision-making at this level. Countries with single-payer systems may be better able to make use of COC as a measure of systems improvement. Under the current system in the United States, having multiple payers creates multiple systems that gather data separately.

In comparing the data collection capacity for the three diseases currently, the standardised HIV system is the most advanced. While HIV has mandatory reporting and standardised data collection, these tools are not available to those seeking to measure systems improvement in addiction services or for treatment of HCV. This means that when it comes to treatment utilisation, each separate payer, whether an insurer like Medicaid or private insurance companies or public programs like the SAPTBG or jail programs, has a separate data file with this information. To compound the complexity, each different type of medication for opioid use disorder has additional separate, non-integrated sources of data. Buprenorphine prescriptions are routinely recorded in prescription monitoring programs (PDMPs) while generally methadone is not. All patients receiving methadone treatment must be registered in a state database to prevent duplicated enrolment, but patients receiving other OUD treatment are not in these registries. Reporting systems that feed into the TEDS system requires that patients appear as “dropouts” from methadone treatment when they transfer from one program to another, even within the same organisation, contributing to an inability to construct accurate retention thresholds. Moreover, in PDMPs, data are generally designed for interdiction and not for addressing the OUD epidemic.

Without an accurate count of individuals with OUD in need of services or currently receiving evidence-based treatment services, states are not able to efficiently deploy resources that have been provided by the federal government to address the overdose epidemic amongst disenfranchised populations. If standardised cascade measures for OUD were agreed upon, continually collected by states, and analysed on an annual basis at the per-person level, states and health systems could effectively plan interventions that provide the most impact for this high-need population. Without them, health systems and treatment providers cannot fully define the burgeoning issue that they are trying to solve and run the risk of ineffectively spending hundreds of millions of dollars annually.

The cascades that could be created did however, point to important findings. For OUD treatment, the data available indicated that treatment entry, or linkage to care, is where there is the largest target for improvement as indicated by the largest change between cascade steps. As for HIV, treatment retention appeared to be the greatest target. With previous studies suggesting that improvements in treatment entry for OUD should lead to improved treatment retention for HIV, this further supports the need for OUD cascade development [12,34].

Overall, the findings from this study show that there is an opportunity to improve data use to address the syndemic of OUD, HIV, and HCV. Though HIV cascades do exist in most states, our findings illuminate that data are lacking for individuals with both HIV and OUD. Unlike HIV, where providers are mandated to report data that address the 95-95-95 goals, there is no standard methodology for tracking OUD incidence, treatment utilisation or treatment outcomes, and no improvement goals that are universally accepted. Without data or targets, states cannot effectively plan for services, nor measure the effectiveness and efficiency of their expenditures. When considering that the federal government – NIH, CDC, HRSA and SAMHSA, earmarked over $456** **M in grant funding for HIV, HCV, and OUD initiatives to these five states alone in 2019, this represents an astronomical data gap [35–38].

## Path forward and recommendations

### New data analyses and frameworks to guide public policy

Data collection and goal setting methods used to combat the HIV epidemic, like the 95-95-95 goals, have been demonstrated to be applicable to OUD and HCV as research activities, but have not been applied routinely as a means for improving outcomes for OUD or HCV. For this to occur, states would need to create all-payer claims database so that the data are centralised, and all systems use the data for planning and assessment. The continued growth in overdose death rates and the reversal of positive trends in infection rates are a clear demonstration that the inadequate ability to measure outcomes has led to ineffective deployment of five years of significant federal increases in funding.

### Targeted implementation strategies to address syndemic conditions

There are four well-researched evidence-based practices to reduce the spread of HIV among people who use drugs: syringe service programs, treatment as prevention (TasP), MOUD, and PrEP [39]. There is community level resistance to all four of these strategies [40,41]. One tool to reduce stigma is the provision of high quality services that PWID will use and local data that demonstrates these successes. In the absences of promising practices supported by local data, states are reliant on research to defend policy choices and the general public is often mistrustful of research on stigmatised issues [42].

### Tools to support policy priorities and decision-making

Even with data, policy makers are unsure how to prioritise spending. While data can support to whom interventions need to be targeted, and can provide feedback on whether the targeting is successful, data alone are not enough. Decision support systems either using local data, or using research-based statistics are key tools that have been used to address other public health crises. Advances in disease modelling techniques have made it possible to predict the expected outcomes of the implementation of certain interventions within populations and should be made available to policy makers to support their systems level interventions.

## Limitations

There are a number of limitations identified while conducting this study that primarily involve the quality of the data. First, OUD cascades cannot be compared to one another due to divergent sources of data, data collection methods and populations for whom data are collected. Therefore, the bar charts should not be seen as a means of comparing states to each other. Without a central database collecting cascade components, we compiled the COC’s utilising data from various sources including national surveys, state level reports, and grant applications. Even if the same reports were used across states, for example 2017 TEDS data for prevalence, the populations of which each state chooses to report could vary. Utilising a general population survey such as NSDUH also has its limitations as certain groups are excluded – homeless persons who do not use shelters, military personnel on active duty, and residents of jails or hospitals [43]. In addition, measures related to opioid use are subject to substantial underestimation by household survey methods [30]. The analysis for each state has missing data either on specific treatments (buprenorphine) or populations (private insurance), so should only be viewed in the context of that specific state’s data collection profile. Lastly, to avoid undue and potentially harmful additional scrutiny we chose not to identify the states in our study. Although additional population level data were provided in Table 4, the potential for overlooking contextual data serves as a limitation in this study.

**Figure 5.**
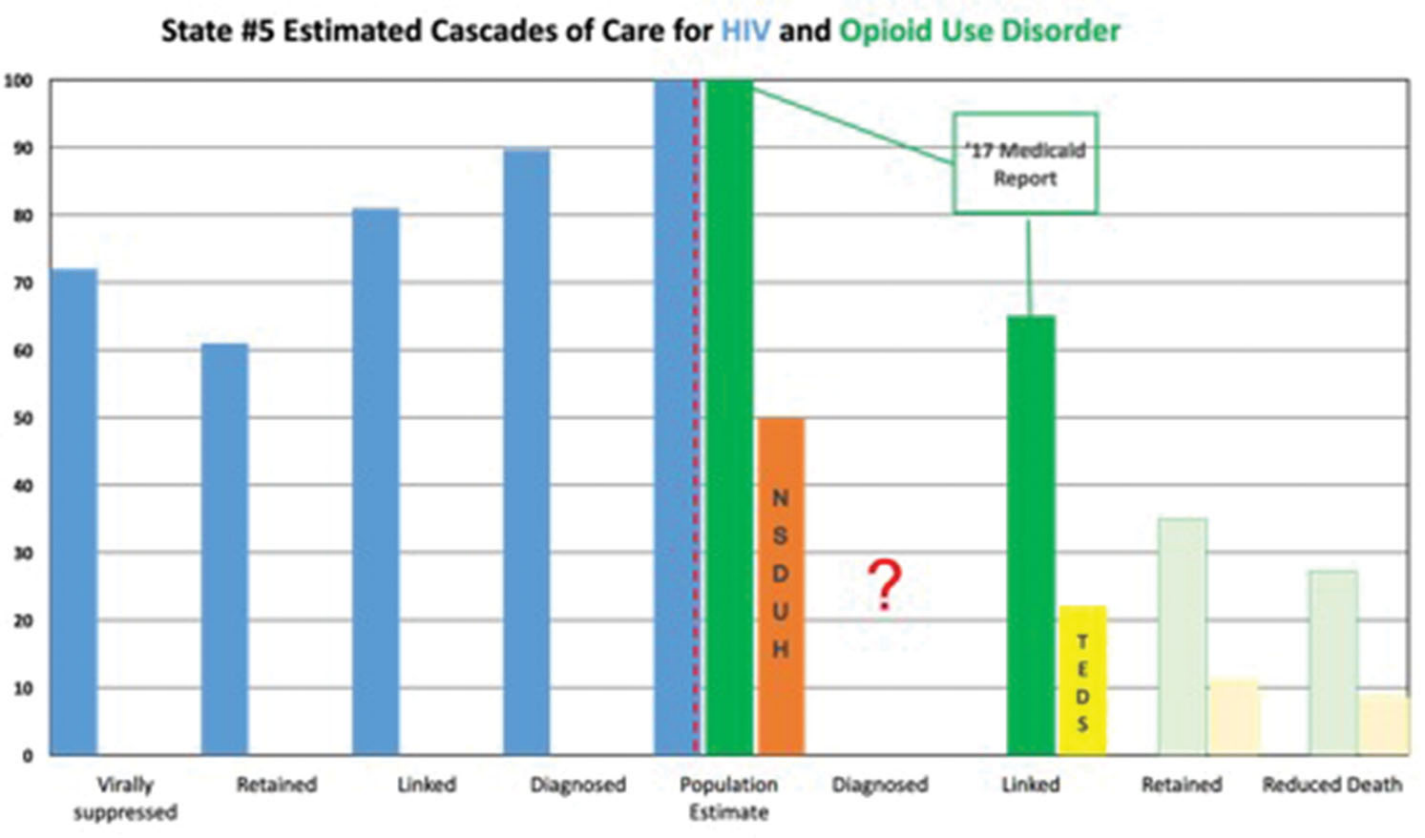
State #5 cascade of care estimates. Axis definitions – “Linked” is equivalent to “linked to care”. “Retained” is equivalent to “retained in care”.

**Figure 5.**
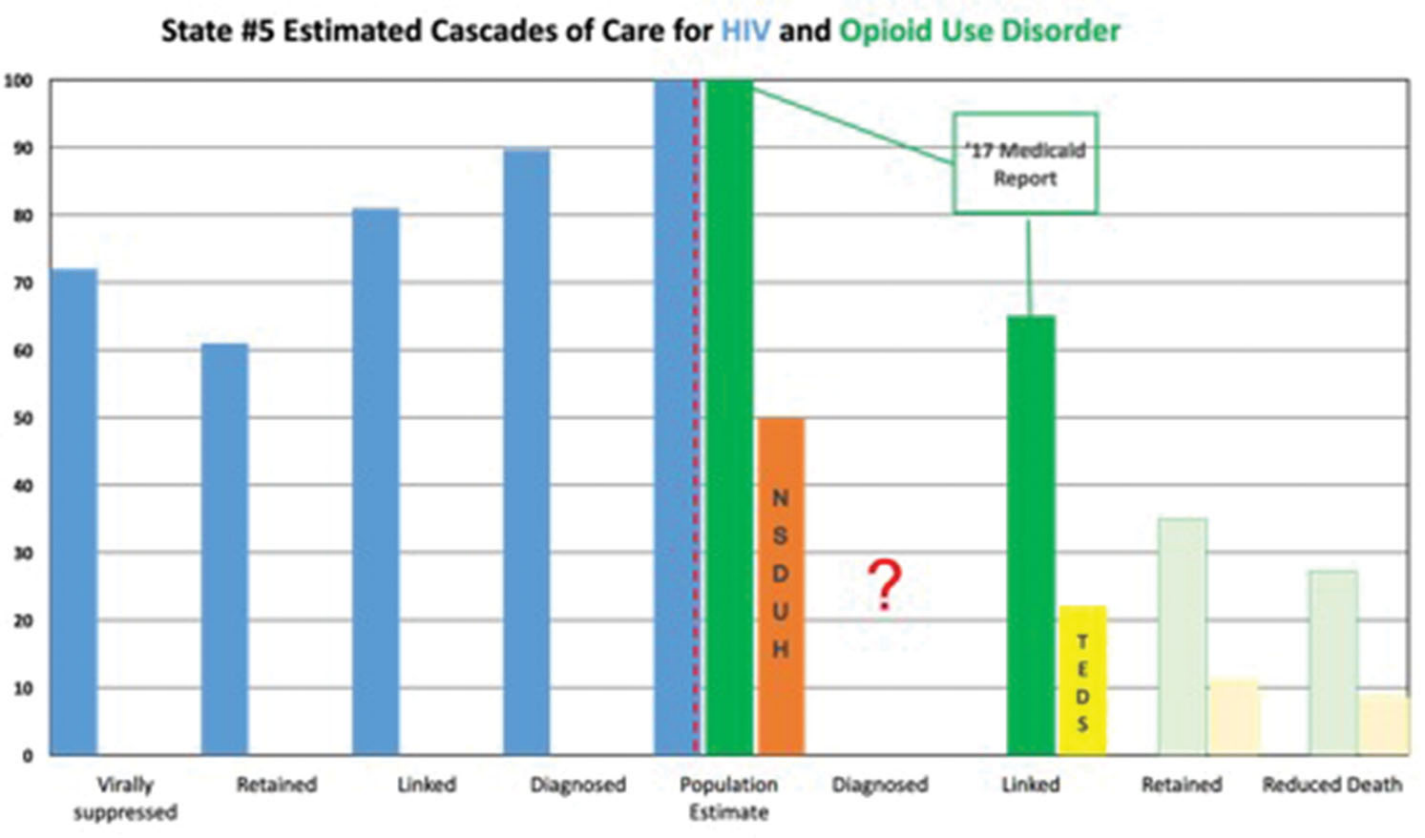
State #5 cascade of care estimates.

**Table 2.** Population estimates.

	States				
Topic	1	2	3	4	5
Region	Appalachia	New England	New England	Appalachia	New England
Total Population 2020^a^	1–2 million	< 1 million	1–2 million	4–5 million	3–4 million
Percentage of Counties Rural^b^	71%	93%	32%	85%	13%
Percentage of population enrolled in Medicaid or CHIP 2020^c^	25–37%	25–37%	11–18%	25–37%	23–25%
	Rate per 100,000 Population				
Prevalence of HIV – Persons living with diagnosed or undiagnosed infection 2018^d^	125–150	125–150	150–175	225–250	350–375
Prevalence of OUD 2017^e^	825–850	1100–1125	1250–1275	1200–1225	550–575
Drug Overdoses 2018^f^	50–60	20–30	20–30	30–40	30–40
Rate of Reported acute hepatitis C 2018^g^	3–4	0–1	1–2	3–4	0–1

^a^Census, 2021 [44], ^b^HRSA, 2021 [45], ^c^CMS, 2021 [46], ^d^CDC, 2021a [47], ^e^SAMHSA, 2017 [27], ^f^CDC, 2020 [48], ^g^CDC, 2021b [49].

**Table 3.** Data sources table.

	Population prevalence	Identification/diagnosis	Treatment initiation	Retained in care	Patient outcome
HIV	CDC	State HIV webpages	State HIV webpages	State HIV webpages	State HIV webpages
OUD	NSDUH, State reports, Federal Reports, Foundation Reports	Not available	TEDS, state reports, federal reports	Research, not available as local data	Research, not available as local data
HCV	CDC/State public health webpages	State public health webpages	N/A	N/A	N/A

## Conclusions

Absent the ability to identify the crucial gaps in care and efficient opportunities to address them, or measure how interventions improve process and outcomes for people with conditions like OUD, states, healthcare systems, and the federal government run the risk of financing poorly targeted interventions that have little impact on relevant patient outcomes. Significant investments by state and federal governments have been made in recent years, yet the overdose rate continues to climb, and HIV and HCV outbreaks continue to occur in regions where OUD is prevalent. Directing some of the investment towards data infrastructure might ensure the rest is used effectively.

## Data Availability

The data that support the findings of this study are available from the corresponding author, AE, upon reasonable request.
